# Co-creating support: a participatory research approach to developing a group intervention program for parents of children diagnosed with cancer

**DOI:** 10.1186/s12913-026-14402-8

**Published:** 2026-03-20

**Authors:** Mélina Rivard, Léandra Desjardins, Zakaria Mestari, Christine Lefebvre, Shaneha Patel, Julie Tremblay, Élodie Bergeron, Juliette Bellenger, Lysiane Roch

**Affiliations:** 1 Département de Psychologie, Université du Québec à Montréal (UQAM), C.P. 8888 succursale Centre-ville, Montréal, Québec,, H3C 3P8 Canada; 2 CHU Sainte-Justine, Montréal, Québec,, Canada; 3 LEUCAN, Montréal, Québec,, Canada

**Keywords:** Pediatric cancer, Community-based participatory research, Acceptance and commitment therapy, Parent psychosocial intervention

## Abstract

**Background:**

Parents of children diagnosed with cancer face significant psychosocial challenges, yet their needs often remain insufficiently addressed within existing services networks. Few interventions documented their clear theoretical foundations or mechanisms of action, limiting understanding of how specific components lead to meaningful outcomes, particularly those valued by parents themselves. To address these gaps, a community-based participatory research initiative was launched by a community paediatric cancer association in collaboration with academic researchers. The current article focuses on co-developing an intervention model to support the well-being of parents whose child has recently received a cancer diagnosis.

**Methods:**

Using an iterative modified Delphi process (series of questionnaires and focus groups), we engaged parents and clinicians to document their perspectives on the essential components of a parent-focused psychosocial intervention. Twenty-one participants (14 parents, 7 clinicians) contributed to this phase of the larger initiative. The mixed-methods data provided quantitative data analyzed using descriptive statistics and qualitative data analyzed using thematic analysis.

**Results:**

This collaborative process led to the development of “Me for Us,” a structured parental group intervention. This manualized, eight-session program was designed to enhance parents’ psychological well-being and their capacity to support their child and family.

**Conclusions:**

Grounded in principles of Acceptance and Commitment Therapy, “Me for Us” focuses on informational resources, self-care strategies, emotional regulation, peer support, and parent–child relationships. Key mechanisms such as small size group, co-facilitation by a mental health professional, and including a parent-partner with lived experiences are discussed.

Receiving a cancer diagnosis in childhood can profoundly impact all family members, creating significant challenges in the daily lives of the child, their siblings, and their parents [1–3]. Parents of children diagnosed with cancer are particularly vulnerable, as they face multiple and cumulative stressors while caring for the child and attending to the needs of other family members [4]. These stressors include uncertainty regarding the child’s treatment and prognosis, disruptions to daily routines, the need to comprehend complex medical information, and the challenge of navigating intense emotional distress, often with limited support available to them [5]. Estimates of the presence of clinical symptom levels amongst parents vary, with a meta-analysis reporting prevalence rates as high as 65% for anxiety, 91% for depression, and 75% for post-traumatic stress disorder [6]. Providing psychological support to parents from the time of diagnosis is essential both to support parents at heightened risk of psychological distress and to promote the well-being of the child through its positive effects on parental adjustment and caregiving [4, 7].

## Gaps in parental support within pediatric cancer services

Supporting caregivers has been established as a psychosocial standard of care since 2015 [8]. Despite the well-documented impacts on parents, caregiver psychosocial needs are often poorly addressed in the context of pediatric cancer [1, 2, 9]. For example, findings from a large U.S. survey suggest that psychosocial support for parents in pediatric oncology remains largely unstructured, with care predominantly delivered through informal discussions (98.2%) or external referrals (93.9%), highlighting a persistent implementation gap in the integration of evidence-based, parent-focused psychosocial interventions within pediatric oncology services [10]. Similarly, in Quebec, the Canadian province where the current study took place, results from a family survey showed substantial variability across regions and pediatric hospitals in the timing, duration, content, and clinical approaches of psychosocial services and highlighted ongoing access, quality, relevance, and implementation gaps, limiting the consistent delivery of evidence-based support to families . Furthermore, parents encounter significant barriers when navigating fragmented service systems to access support for themselves [4]. These difficulties are often exacerbated by long wait times and a general lack of awareness among community clinicians regarding the unique challenges associated with pediatric cancer and its broader impact on caregivers and families. There is thus a clear need to develop and implement accessible and structured psychosocial interventions to address the unmet needs of caregivers of children with cancer early in their care trajectory, as evidence-based mental health practices for parents do not yet appear to be accessible and systematically integrated into routine care.

**Fig. 1:**
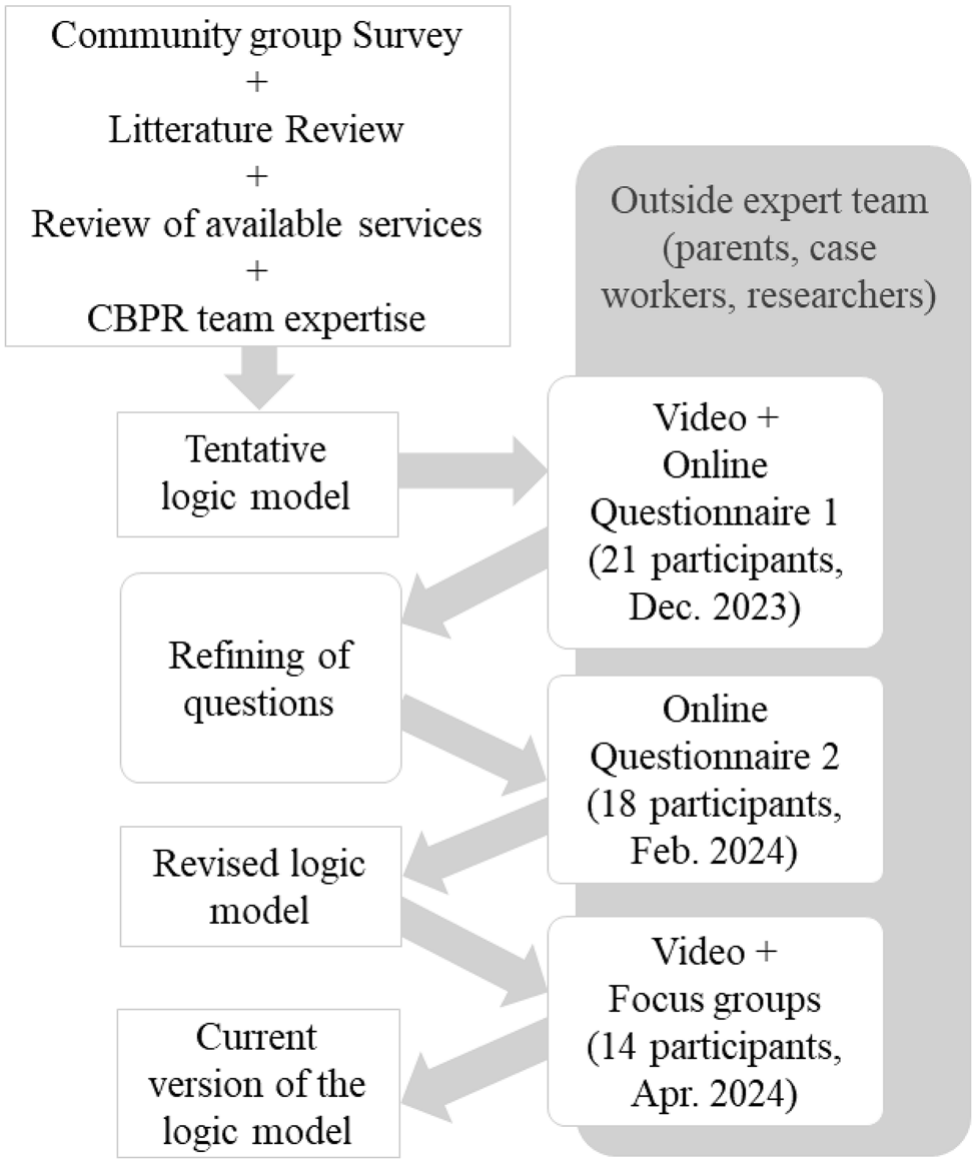
Procedure to develop the logic model

Considering the literature on research-supported interventions, although various psychosocial programs have been developed to support parents, few are specifically designed for the early diagnosis and treatment period, an especially vulnerable time for families. Notably, even fewer interventions are grounded in parents’ and clinicians’ input, particularly the perspectives of those who receive and deliver the support. The voices and lived experiences of families and clinicians are often missing in both the intervention design and evaluation phases, leaving essential questions unanswered about how to best tailor interventions to meet their actual needs [2, 11]. Additionally, few programs clearly outline their theoretical foundations or mechanisms of action, making it difficult to understand how specific components lead to desired changes [2]. Moreover, there is limited information on how the content of interventions has been selected, how therapeutic approaches and strategies have been chosen, and how outcomes have been identified and targeted, particularly the outcomes that parents themselves view as most meaningful. Key aspects such as program length, individual versus group formats, facilitator qualifications, and optimal timing within the family’s cancer journey vary widely across initiatives. Furthermore, very few programs are developed for delivery within community-based services or adapted to the resource constraints and realities of these settings. Engaging knowledge users, including parents and community service providers, in the co-development of interventions from the outset could help bridge these gaps and facilitate the integration of evidence-based mental health practices into routine care [12].

### Enhancing family psychosocial services: co-development of a parent group intervention

To address existing gaps and develop accessible and socially valid psychosocial interventions for family members in context of pediatric oncology, a large initiative was launched by a community pediatric cancer association in collaboration with academic researchers with the aim of: (1) better understanding the unmet psychosocial needs of family members (see ); (2) collaboratively designing comprehensive programs that align with these needs, involving families, practitioners, and researchers throughout the process; and (3) jointly evaluating these programs as they are implemented within the routine services of a community-based pediatric cancer organization. This large initiative is grounded in the principles of community-based participatory research (CBPR; [13]), which conceptualizes community knowledge users as co-researchers sharing responsibility for defining research priorities, guiding methodological decisions, and interpreting findings in studies aimed at developing, enhancing, and implementing supports to improve access to and quality of services for populations experiencing social and health inequities [12, 13]. CBPR guiding principles include valuing community expertise, building upon existing strengths, and promoting shared power and resource allocation across all phases of the research process. CBPR further seeks to balance scientific objectives with tangible community benefits, foreground community-identified priorities, and requires sustained engagement that extends beyond discrete funding cycles, with an explicit emphasis on capacity building [13]. By engaging both users and service implementers in the decision-making process for research and interventions, this approach enhances the social relevance of the interventions and fosters sustainable changes in practice.

For this large CBPR initiative, an advisory committee was established. The committee comprised academic researchers (professors and graduate students), clinicians, an administrator from the community-based association (Leucan), and a parent. Acting as equal co-researchers, committee members were tasked with guiding and coordinating the CBPR initiative and defining research priorities. They determined which studies and intervention development projects to initiate first, with the plan to begin with one intervention project and gradually expand to additional projects over time. The advisory committee used the results of a large survey conducted with 441 family members of the community pediatric cancer association to pinpoint the highest priorities for psychosocial interventions as experienced by families (Rivard [14]). One of the top priorities identified was the need for a comprehensive intervention program to support the psychosocial well-being of parents following the child’s pediatric cancer diagnosis. Specifically, parents reported a need for support with coping with emotions (76%), dealing with the psychological impacts of the diagnosis and prognosis (69%), managing mental load (67%), and coping with fear of cancer recurrence (66%). In addition, most parents reported having received only sporadic psychosocial support, usually through isolated consultations with professionals such as psychologists or social workers. Many expressed a strong desire for a more structured and comprehensive program, one that supports their own psychosocial well-being, but that also equips them to better support their child’s emotional journey. This need was identified as especially urgent during the early active treatment period, which parents described as a particularly overwhelming and vulnerable time.

As such, the co-development of a psychosocial intervention for parents that could be delivered via the community pediatric cancer association was selected as the first interventional research project. Consistent with CBPR principles, all advisory members were actively involved throughout all phases of the research process, including the formulation of project objectives, identification of guiding principles to develop the research design and intervention, and validation of research outputs. To address gaps in the literature, the team prioritized co-developing the parental intervention with input from both parents and clinicians and a co-development design that would allow a clear description regarding how intervention content, theoretical foundations, mechanisms of action and therapeutic strategies, as well as intended outcomes are selected (see the detailed co-development design in the method section). Moreover, the iterative process to develop the intervention was consigned and anchored in the implementation science concept of a logic model, which provides a structured representation of an intervention by articulating its core components, underlying rationale, and the relationships between resources, activities, and outcomes (Chen, [15]; [16]). The logic model of an intervention typically specifies the problem addressed, target population, required resources, activities, outputs, and short-, intermediate-, and long-term outcomes, while also accounting for contextual factors that may influence implementation. Importantly, logic models are dynamic tools that can be developed or refined throughout a program’s lifecycle, supporting adaptation and long-term sustainability in complex and evolving service settings. By mapping how inputs are transformed into processes and outputs within a given context, logic models guide program planning, implementation, and ongoing evaluation.

### Objectives

This first intervention development study of the CBPR initiative aimed to develop and refine the logic model of a psychosocial parental intervention with input from knowledge users (parents of children diagnosed with cancer, clinicians, researchers, and community organization psychosocial providers) through a modified Delphi process [17]. As such, this article presents the results of the three-step process (two rounds of questionnaires to build the logic model and one series of focus groups for its validation), undertaken to identify the key components of the new program (problems/needs, goals, targeted population, resources, activities, outputs, outcomes, and contextual factors). This logic model was the foundation for developing the program manual and materials for the final program “Me for us,” which supports parents through informational resources, self-care strategies, emotional regulation, and strategies to improve parent–child relationships.

## Method

This study was approved by the ethics review board of the University (Redacted for blind review). The co-construction of the intervention logic model relied on an adapted Delphi procedure in three stages (a first questionnaire based on literature and the Quebec psychosocial needs survey, a second questionnaire to refine the key components of the logic model and the last stage of restitution of the model through focus groups). This methodological integration was designed to promote convergence across knowledge user perspectives while ensuring the contextual relevance, feasibility, and social validity of the resulting model. The advisory committee members led the development of the tools (e.g., questionnaires, focus group guide) and the iterative analysis at each stage. Across the three stages, the advisory committee members used a data extraction grid to categorize information according to the components of a logic model (e.g., resources/inputs, activities, influencing factors; Chen, [15]). This grid was applied to identify and organize relevant information and refined throughout the analytical process. The contents of the logic model integrated data triangulated from the various sources and were systematically discussed at each stage among team members. This method has been used in several co-production initiatives with public agencies in the Quebec Health and Social Services by our research team and the process itself has been described extensively in a methodological paper (Rivard et al., [18]).

### Participants in the modified Delphi process: 21 knowledge users

The data extracted at each stage came from the input from 21 knowledge users (20 women and one man), of which 14 were parents and 7 were clinicians in pediatric oncology (Table 1). Our sample size is aligned with the recommendations of Beiderbeck and colleagues [19] regarding the number of participants required to constitute a Delphi panel (a minimum of 15 to 20 participants). Parents were recruited through a monthly newsletter of the partnered pediatric cancer association emailed in November 2023. The advantages of this recruitment strategy were threefold: (1) to reach the largest possible number of parents; (2) to reduce the recruitment burden for the association’s practitioners; and (3) to ensure consistency with the recruitment procedures used in other research projects involving the association. No disadvantages for the target population were identified by the advisory committee regarding this recruitment method, although the research team acknowledged that a more personalized approach might have resulted in higher participation rates among parents. Clinicians were contacted by email. Both parents and clinicians were provided study information which included a description of the project, its requirements, and the eligibility criteria. Participants who expressed interest in the project were sent the consent form in December 2023.

**Table 1:** Modified Delphi process participant characteristics

	*N*	%
Gender		
Male	1	4,8
Female	20	95,2
Parents (*N* = 14)		
Child diagnosis		
Leukemia	9	64,3
Cerebral tumor	4	28,6
Burkitt lymphoma	1	7,1
Treatment		
Chemotherapy	14	100
Transplant	6	42,9
Surgery	4	28,6
Radiotherapy	3	21,4
Other	1	7,1
Treatment stage		
1–2 years post-treatment	2	14,3
2–5 years post-treatement	7	50
Long-term remission	4	28,5
Bereaved	1	7,1
Clinicians (years of experience = 2 to 18, M = 7,9)		
Role		
Social worker	3	42,9
Support worker	2	28,6
Special educator	1	14,3
Psychologist/pediatric cancer research	1	14,3

The inclusion criteria for parents were: (1) being the parent of a child who had received a pediatric cancer diagnosis, (2) being a member of the participating community pediatric cancer association and (3) having the ability to understand and communicate in French. Among the 14 parent participants, there were two who had children 1 to 2 years post treatment, seven who had children 2–5 years post treatment, four who had children in long-term remission, and one bereaved parent. Child diagnoses included: leukemia (for nine parents), cerebral tumor (for four parents), and Burkitt lymphoma for one parent. The treatments received included chemotherapy (14 children), transplant (six children), surgery (four children), radiotherapy (three children), and other targeted pharmaceutical treatment (one child).

The clinicians in pediatric oncology included three social workers, two support workers, one special educator, and one psychologist with research experience in the field of pediatric cancer. Clinicians worked in the domain of pediatric oncology for on average 8 years (*Range* = 2–18 years).

### Procedure and instruments: the three stages of the co-creation of the logic model

The adapted Delphi process unfolded over three iterative cycles, combining features of classical Delphi methods with qualitative data collection and collective deliberation. The classic Delphi method is a structured consultation technique designed to achieve consensus among a panel of experts on a given topic. Traditionally, this method involves four iterative rounds of consultation [20], although the number of rounds may vary depending on the nature of the topic and the level of consensus sought. A modified Delphi process represents an adaptation of the classic approach to better align with research and practice contexts. Such adaptations may include reducing the number of rounds and incorporating mixed data collection strategies (e.g., the inclusion of open-ended questions or focus groups to support majority-based decision-making). These methodological adjustments aim to maintain acceptable response rates in contexts characterized by limited participant time and capacity, reduce time and resource burden, and facilitate efficient decision-making in collaboration with knowledge users, particularly when decisions are complex and unlikely to achieve unanimous agreement [14]. Figure 1 presents a visual summary of the mixed-method procedure to co-construct the intervention logic model.

### First stage: initial logic model creation

In order to create the first questionnaire, the advisory committee first created a version of the intervention logic model with different possibilities of intervention characteristics/features based on the following sources of information: (1) the results of the survey with Quebec families on the need for mental health supports; (2) a portrait of existing services in Quebec (in order to avoid investing in an intervention whose mission is already met by existing services); (3) clinical recommendations and guidelines available in the scientific literature (grey literature search assisted by a specialized librarian); and (4) the expertise and lived experience of our advisory committee.

A questionnaire was created under the principles of co-production and several consensus meetings between team members. It was pre-tested with one doctoral candidate in psychology, one parent, one clinician with a Master’s degree in psychoeducation, and the community organization manager. Questions were intentionally formulated to cover the core components of an intervention logic model. The questionnaire was divided into four sections: (1) socio-demographic data, (2) elements related to the program structure and delivery methods, (3) objectives and session content, and (4) mechanisms of action and intervention. The first section included multiple-choice questions to provide a profile of the participants. The second section primarily featured open-ended questions to gather participants’ perspectives, along with a few multiple-choice questions about the delivery format. The third section involved ranking questions with open-ended comment options (topics to cover) and open-ended questions. The fourth section consisted solely of open-ended questions.

The first round of the questionnaire was completed by participants in December 2023. Before completing the first questionnaire, participants were introduced to the initial version of the logic model of the intervention through an online video, accompanied by a diagram illustrating the model. This allowed participants to review and familiarize themselves with the content before engaging in the questionnaire.

The data of the 21 completed questionnaires were then analyzed (as detailed in the Analysis section), which facilitated the development of the second questionnaire in the modified Delphi design. This analysis helped identify key themes and areas where further clarification or consensus was needed, guiding the formulation of the questions for the second round.

### Delphi questionnaire round 2: second online questionnaire

The second round of the Delphi process consisted of a questionnaire based on the analysis of the responses from the first questionnaire responses and was designed to allow the research team to either validate and confirm consensus on aspects of the program that had been endorsed by the majority of participants, gather more specific details on aspects that required further clarification to reach a consensus, or introduce new questions related to the logic model that emerged from the qualitative analysis. The online version of the questionnaire was also pre-tested with two doctoral students in psychology, two undergraduate students in psychology and a parent. The questionnaire followed the same three main sections as the first questionnaire, excluding the socio-demographic data.

For the second round of the Delphi method, the 21 participants of the first round were contacted via email, which included the link and explanation for this new step of the study. The second round was completed by 18 participants from the first round (1 researcher, 6 clinicians and 12 parents) in February 2024. After receiving the 18 completed questionnaires, the data was analyzed to update and finalize the logic model of the intervention and prepare the interview guide for the Phase 3 focus groups.

### Restitution of the data and validation of the logic model: focus group

Finally, participants who responded to the first two rounds of the Delphi process were invited to take part in focus groups in April 2024. In total, three groups were formed: one group consisting of five clinicians, and two groups made up of four and five parents, respectively. A total of 14 participants took part in the focus groups.

During a virtual focus group, which lasted approximately one hour, participants were invited to validate and share their opinions on: (1) the target population, (2) the program objectives, (3) the components of the intervention (group, type of facilitation, and materials), (4) the content and structure of the sessions, (5) the mechanisms of intervention, (6) the expected outcomes; and (7) perceived barriers and facilitators. A member of the research team acted as facilitator to encourage balanced participation among the group members and ensured that all topics were covered. The audio recordings of the meetings were subsequently transcribed verbatim by a research assistant.

### A last phase of integration of the data to finalize the logical model

The advisory committee members employed a data extraction grid to systematically categorize information based on the components of a logic model. Specific criteria were employed to select final intervention components : (1) majority in the responses of the three stages modified Delphi; (2) integration of qualitative data ensured that the final research outcome accurately reflected new participants’ insights and contributions; (3) remaining aligned with the overarching goals initially established by the advisory committee members (e.g., accessible for all families in Quebec, feasible for the community association). This approach acknowledged the team’s expertise in making final decisions on interpreting which intervention features to include in the final logic model using both quantitative and qualitative data. As such, the final logic model was finalized by the advisory committee for collective validation. Feedback was discussed and integrated through an iterative process until consensus by convergence was achieved, rather than through quantitative agreement thresholds (see [21]).

### Analysis

**Online questionnaires (two first stages)**. Quantitative data were analyzed using descriptive statistics (frequency and percentage). In accordance with the recommendations of Diamond and colleagues (2014), for the quantitative data, consensus items reaching majority agreement were retained in the model. Open-ended questions were analysed using the structured tabular thematic analysis method (ST-TA; [22]). ST-TA involves the following steps: (1) repeated readings of the entire dataset to become acquainted with the data, (2) assigning initial codes to each segment (or units of meaning), (3) generating themes and subthemes (4) categorization of all verbatim transcripts, and (5) calculate the number of respondents for each theme and subtheme. Inter-rater agreement made on 100% of the material by two different coders trained in qualitative analysis was excellent (> 95%).

**Focus groups**. The method proposed by Rabiee [23] was used to analyze focus group transcripts. This approach adapts the Ritchie and Spencer [24] method to the focus group format, while incorporating the Long Table technique from Krueger and Casey [25] to process the emerging qualitative data from participants’ discourse. According to this method, researchers are first encouraged to familiarize themselves with the material by listening to the recordings and reading the transcripts. The transcripts are then printed on different colored paper for each group. When participants provide a direct response to a question, the meaning unit is identified and categorized into the appropriate pile corresponding to that topic. If participants introduce a new topic, the meaning unit is separated and placed in a new pile, creating a new theme. Next, the researchers collaboratively review the meaning units within each theme pile to generate sub-themes or reassign the meaning units to more appropriate themes. This method allows for the calculation of frequency (the number of participants who mentioned a theme), extent (the proportion of participants who mentioned a theme), and consistency (the representation of a theme across the different groups).

## Results

### Results of the two questionnaire rounds

The results of the two Delphi questionnaire rounds are presented consecutively by components of the logic model.

**Program Objectives**. Participants of the first questionnaire round (21 participants) were invited to provide their opinion on the following objective: “To teach and put into action competencies to promote the psychological well-being of parents in the context of pediatric cancer.” All participants (*N* = 21; 100%) were in favor of the initial objective. To expand on this objective, participants were asked questions on the topics to cover during program sessions. More specifically, they were asked to rank, in priority, the following spheres mentioned in the survey: Personal, family, conjugal, and social (see Table 2). During the second Delphi round (18 participants), 100% of participants agreed on prioritizing the personal and family spheres, particularly the parent-child relationship. Moreover, 17/18 participants (94%) indicated that they agreed to remove the conjugal sphere of the program (financial and couple). To this effect, a participant mentioned, “This could be a delicate subject for some. Certain parents that I knew were separated, single, and in the divorce process, mourning the loss of a partner, so this could be a topic that does not concern certain parents.” The financial sphere was removed from the program because formal supports are offered for this aspect in the partner-community setting.

**Table 2:** Themes to address in the program sessions

Sphere	Sub-theme	Mean rank	SD	Example quotes
Personal Sphere	**Stress/Anxiety**	**2** **,2**	1,3	“Stress related to treatment side effects and accumulating fatigue, financial stress, stress of losing their child/of a relapse or disease progression.”
	**Psychological and Emotional Distress**	**3**,**1**	1,2	“When the diagnosis comes, we go through the stages of grief, grieving the loss of seeing our child grow up healthy, the grief of not being able to protect them from the suffering they will face. Our world collapses in a fraction of a second.”
	Trauma	3,8	1,7	“Fear of treatments and their effects, fear of isolation, loss of bearings, fear of death, fear of losing their place in their environment, fear of relapse, fear of physical appearance, self-esteem, fear of lasting effects.”; “Then, explain the terms, for example, many parents say they are in post-traumatic shock…”
	Feeling of Uncertainty	3,8	1,6	“Having to live a life where it’s impossible to truly plan, not knowing where we’re headed… It’s one surprise after another, we’re often on an emotional rollercoaster, not really knowing if we’re on the right path. Coping with pediatric cancer feels like walking a tightrope, we’re constantly afraid of falling off… this unpredictability of what lies ahead (there’s no real game plan) is related to the possibility of not making it out alive…”
	**Mental Load/Parental Burnout**	**3**,**5**	2,0	“The mental load becomes enormous in the context of pediatric cancer. The parent who stays at the child’s bedside takes on even more roles than before: they become a doctor, nurse, friend, parent, confidant, cook, housekeeper, the one who receives all the emotions… Despite all this, the parent is unable to take time for themselves, to pause, or to feel their negative emotions, because they know they must remain strong, cheerful, and reassuring for their child. When treatments end, parents often ‘crash’, they collapse, because all this catches up to them.”
Family Sphere	**Behavior Management**	**3**,**0**	1,7	“Understanding the needs hidden behind behaviors… parents no longer know how to set limits for their sick child after the diagnosis. It’s hard to say no and put limits with a sick child… sometimes parents are confused and helpless in front of their child’s behavior, often caused by medication. They wonder if they should intervene, and especially how to do so?”
	Challenges Related to Life Transitions	5,0	0,9	“Once again, throughout the trajectory, all of this can change. For example, if another child dies in the hospital and our child becomes aware of it… We need to learn how to communicate these things to our child without causing further trauma.”
	**Challenges in Family Organization**	**2**,**8**	2,0	“Managing hospitalizations, other children at home. Technical management of all the claim forms, school follow-up, work, etc. Reorganizing respective schedules of each family member. Managing the emotions of all family members.”
	Challenges Related to Changes in Family Relationships	4,1	1,7	“One of my greatest difficulties was the pressure I felt from family members (my son’s father and grandparents) to reassure them, while I was anxious myself… I already had to be there for my son, be his pillar, and acting as a psychologist for the rest of the family was too much for me.”
	**Challenges related to the Affected Child’s Mental Health**	**2**,**9**	1,5	“Verbalizing one’s emotions/anxieties… so how to be present for the child, how to help them manage their emotions and make them feel safe. Parents should have tools to be able to address the situation directly with their child, to be able to support them.”
	Challenges Related to Sibling’s Mental Health	3,6	1,4	“Parents often report feeling guilty for not paying attention to the siblings and their emotions while they are caught up in the whirlwind with the affected child; priorities overlap here as well.”
Conjugal Sphere	Challenges Related to Financial Aspects	1,5	0,6	“I believe that financial security can be an aggravating factor for many families, especially in the current situation where inflation is at its peak. Some families are already struggling financially, and a serious illness diagnosis can further jeopardize their financial stability.”
	Challenges Related to Changes in the Couple’s Relationship	1,7	0,7	“Often, one parent takes on the entire mental load of the illness aspect. There’s a feeling that the other parent is less competent or less involved. The other parent may be less informed, [this] may minimize or amplify the situation.”
Social Sphere	Challenges Related to School Issues	2,4	0,9	“If the child is attending school, how can we reintroduce a routine/a life as close to normal as possible, where they can learn at their own pace and meet other youth.”
	**Challenges Related to Changes in the Social Network**	2,3	1,0	“Parents often describe living in a dichotomy from the rest of society.”; “Losing relationships because you’re never available, because others don’t know what to say to you, because you have nothing fun to share — lack of recognition from society regarding the trauma experienced, including from the family’s own social circle.”
	**Challenges Related to Healthcare Settings**	1,6	0,7	“Not feeling like they have the skills… Not always being considered as care partners who evolve over time… Feeling stripped of their ability to properly care for their child make the right decisions. They feel incompetent, powerless. The healthcare setting is, by definition, alienating for a family; it’s not a natural environment for them, and they often feel like outsiders. Families don’t know the inside of the healthcare system, and many things can seem absurd to them. The intrinsic vigilance of some families may be misinterpreted… the way some families express their anxieties can also be very poorly received.”

* Priorities (1 = Highest priority) based on Round 1; themes chosen for the second questionnaire are in bold text

During the two rounds of questionnaires, participants indicated in the open-ended question (qualitative analysis) that the following themes should be covered in the program: (1) Fears, uncertainty, and feelings of helplessness in the face of remissions, after effects and the death of a child (R1: *n* = 6; R2: *n* = 18); (2) Coping strategies and empowerment to face challenges and regain control of their lives (R1: *n* = 5; R2: *n* = 18); (3) Managing feelings of guilt and parental worries related to siblings (R1: *n* = 1; R2: *n* = 18); (4) Better defining parental expectations regarding the behavior of the sick child (R1: *n* = 1; R2: *n* = 18); and (5) Setting limits in managing questions and concerns of extended family (R1: *n* = 1; R2: *n* = 5). Five participants indicated that the priority of themes could evolve according to the stage and progression of the illness, thus it is necessary to offer a range of tools to cover the vast needs of families and adjust to the group in consequence: “These priorities must be reviewed according to the parents and especially according to where they are in the typical roller coaster of pediatric cancer.”

**Target population.** During the first Delphi round, through open-ended questions, participants were able to comment on the characteristics of the parents to be included in the intervention. This was then categorized into three themes: inclusion criteria, exclusion criteria, and principles to be followed when creating parent groups. Table 3 details the subthemes within each of these criteria and principles. During the second questionnaire, participants reported agreeing with these criteria and principles, but five participants (28%) nevertheless stated that this type of support should be accessible to all families. Following participants’ comments on the importance of properly dividing parents into groups based on children’s ages, participants in the second Delphi round were asked about the appropriateness of the following division: early childhood (0–5 years); childhood (6–12 years); adolescence (13–17 years); and young adulthood (18 years and older). All participants were in favor, although two individuals mentioned that the distribution should remain flexible, depending on families’ situations and the capacities of service providers.

**Table 3:** Synthesis of the Delphi process round 1 (online Questionnaire)

Themes		Subthemes	*N*	Example quotes
Population: Parents of Children dx Cancer	Inclusion criteria	Psychological availability to participate in a group	8	“Person with the psychological capacity…”
		Motivation to engage in a group	8	“Motivated people to allow others to benefit and not burden the group for other parents”
	Exclusion criteria	Death of child	5	“In my opinion, a parent who has experienced the death of their child following a serious illness diagnosis may have stronger emotions …”
		Palliative care	4	“Parents whose child is at the end of life or incurable may not relate to the concerns of other parents? Could they feel a sense of injustice? Will that scare other parents?”
		Psychiatric problems and substance abuse	4	“Exclusion: Parents who visibly exhibit narcissistic, antisocial, or borderline personality traits. Parents with evident substance abuse problems who would be under the influence during sessions.”
		Risk factors	4	“Exclude if the person is verbally aggressive or otherwise disrespectful.”
	Principles for group composition	Inclusive participation (e.g., Origin, languages, types of families)	5	“Ensure that the participation of racialized people, of different ethnic, cultural, and religious backgrounds (notably First Nations) can be supported.”; “I believe that single parents may also have very specific needs that should be addressed: increased difficulty staying at bedside, siblings can’t be left alone, isolation in serious decision-making, etc.”
		Same stage of treatment and prognosis	5	“I believe that those whose child has relapsed versus those whose child is undergoing treatment toward a ‘first true remission’ probably should not be in the same group. Same thing for parents of children for whom there are no more treatment options (in palliative care), should have separate sessions. These three categories of parents are at completely different stages and have different needs…” “Parents who are just beginning the process, shortly after diagnosis, should also be separated.”
		Validate appropriateness of parents’ needs	4	“In my opinion, a parent who has experienced the death of their child following a serious illness diagnosis may have more intense emotions than a parent who hasn’t gone through the same experience. This doesn’t mean that I believe they should be excluded or separated, but rather that these aspects should be taken into consideration in this study.”
		Varied socioeconomic status levels	4	“Parents with serious financial difficulties might also have specific needs that they would feel ‘ashamed’ to share.”
		Parents of children of different ages	3	“The age of the children could also be considered. The reality between a 15-year-old teen and a 2-year-old child is not the same.”
		Possibility of including other significant adults (e.g., grandparents)	2	“Open to grandparents who are having a hard time, but ‘from a distance’, but who are involved, sometimes as much as the parents.”
		Places of residence/services in the region	2	“Take into consideration isolated families (no family, few or no friends), families coming from far away, and families with multiple children.”
		Equal number of men and women	1	“Include men and women.”
	Participation of two parents in the group	Allow all parents/family types	6	“Should not exclude some families, such as single-parent families or those with little support from the second parent.”
		Respect that some parents prefer to participate alone	5	“Could make it difficult for a parent to share and have their own experience in the presence of the other parent.”, “Being in different groups would allow space to be oneself, to speak openly…”
		Let families decide	5	“I believe it depends on several factors that are personal to each participant (…).”
		Challenges of both parents being available	5	“I don’t think it’s realistic (…).”; “It’s much easier to free up just one parent; it needs to be accessible and simple to participate.”
	Transdiagnostic group			
		Focus on moment of diagnosis announcement, stages, intensity, and impacts of illness, not only on the diagnosis	10	“Group composition should be centered more on similarities regarding the stage and intensity of illness (rather than just the diagnosis itself).”
		Psychological challenges and impacts are the same regardless of diagnosis	9	“I believe that parents who share the same pain, regardless of the cause, can learn from each other (…).”
		More realistic for services to include a variety of diagnoses	4	“Since some types of cancer are quite rare, it would be difficult for parents to join a group, so I think the transdiagnostic component is interesting. I’ve received feedback from bereaved parents saying that the shared experience, though different, is still the cornerstone of their appreciation for bereaved parent support groups, so it seems to me that it could be the same in the context of this project. I believe this practice could be beneficial.”
		Differences enrich exchanges and foster mutual support	5	“Less comparisons when diagnoses vary.”
		Focus on shared support needs	2	“Can help support actions more targeted toward common needs.”
		Focus on grouping by developmental stage/age	2	“Groups should be reflected based on the children’s age groups”
		Help gain perspective on one’s situation and feel less alone	1	“Seeing that some have it ‘worse’ or ‘better’ than us… it can bring hope and comfort when we see parents whose child is doing better/in remission. Same thing for children with more advanced cancer; we might think that if those parents are managing to support their child, then we should be capable as well.”
Facilitator dyad	Co-facilitation by clinician and parent	Complementary expertise, strengths, and limitations	18	“Balancing knowledge based on theory and experience in the program.”
		More positive experience for parents		“Improves the sense of safety, trust, feeling understood, the ability to relate, greater willingness to share, less formal atmosphere.”
		More natural, fluid, and coherent program	8	“It’s important, combining the professional (expertise) with lived experience (the parent) makes it possible, in my view, to address the full bio-psycho-social picture. No matter how much of a professional we are, we haven’t lived it ourselves, and some emotional dimensions may be unintentionally overlooked. The parent can ‘bring us back to reality’ if that happens. Also, for participants, this creates a more natural feeling of understanding and trust.”
		Meets parents’ needs/contact with other parents		“We often receive requests from parents who would very much like to connect with other parents who are living their reality.”; “”Meets parents’ needs to be in contact with other experienced parents without falling into the risk of peer-support groups.”
Online Delivery	Obstacles	Difficulty creating exchanges/fluid participation online	9	“Personality. Some people take up a lot of space, others very little.”
		Difficulty creating a warm atmosphere	6	“Less warm. Can be more intimidating.”; “ Exchanges are certainly less warm at the beginning, and it may take more time to build connections.”
		Technological difficulties	4	“Not everyone is comfortable with technology, and that could prevent some people from joining.”
		Challenges of participating from home	3	“Lack of privacy and being overheard by others at home.”; “Distractions at home.”
	Facilitators	Offer technical support	10	“Have someone available for technical support (…)”
		Offer animation Flexible/dynamic/effective	3	“(…) Dynamic and highly skilled group facilitation”
		Keep microphones and cameras on	3	“Microphone and camera on.”
		Clear guidelines	3	“Put rules in place to ensure the meetings run smoothly.
		Different timeslots	2	“Offer different time slots.”
		During individual meetings, address anticipated challenges and reflect on solutions together	2	“Discuss the best private spot in the house.”
Structure	Preliminary individual meeting	Determine the program’s suitability and parents’ needs	5	“I think it’s a good idea to assess the participant’s needs. There may be cases where a parent is in too much distress to attend group sessions—individual follow-up might be more appropriate…”
		Assess parent’s psychological health	4	“Psychological assessment tools should be used.”
		Clarify expectations toward parents and from parents regarding their involvement	2	“It seems important to me to hold one or more preliminary meetings for this type of research, after having first established clear participation criteria. At this stage, it also seems essential that the parent be able to express their expectations regarding their participation in the research, so that they understand what it entails on different levels (e.g., time commitment, emotional involvement, potential questions/anxiety the research might generate for the parent, etc.).”
		Orient parents toward appropriate services	2	“Preliminary meetings are pertinent; this way, the participant will be oriented to the best resources.”
		Individually describe the child/family’s situation	1	“Also validate in this first meeting whether the parents have concerns about the psychological health of their sick child and/or their other children and help refer those issues to the appropriate professionals.”
Protocol in the case of distress situations		Follow-up with the community organization (e.g., stakeholder to stakeholder)	21	“Being in contact with the organization’s counselors to inform them and notify them of the different steps so that they could free themselves quickly if these families reach out to them.”
		Individualized follow-up and availability between sessions	14	“Ensure individualized follow-ups and personalized and ongoing communication with each parent throughout the program”; “ensure personalized help between sessions.”
		Referrals to other possible resources and building links with partners	14	“Important that parents are referred to other possible resources.”; “Workers involved in family services should be in communication with program stakeholders and informed about the parents’ situation.”
		Informing about the protocol in advance	10	“Describe from the beginning the actions to take in the case of significant distress and share this protocol at the beginning of the program with parents and those concerned.”
		Contact with a significant person	1	“Maybe consider the possibility of including a contact with a significant person, agreed upon at the beginning. Spouse, parent, friend…”
Approaches		ACT	8	“ACT therapy adapted to psycho-oncology because it helps the patient accept their emotions and negative thoughts in order to move toward what truly matters to them.”; “I’m really happy to read this. ACT therapy was really helpful for me. The use of coping strategies and the development of resilience are necessary in this context. I also think that briefly talking about self-compassion could help.”
		Different approaches	5	“‘Draw’ on certain components of theories to apply them to different content, but I believe it would be useful to diversify the approaches.”
		Problem-solving	5	“Since parents already have a lot on their plate, problem-solving therapy might be more appropriate at first…”
		Yoga and meditation	2	“…Anti-stress yoga”; “Meditation. I don’t have personal experience, but it seems to work.”
		Art therapy	1	“Art therapy is a subtle way for parents who have difficulty expressing themselves.”

N = Number of participants that shared information on this theme

### Program delivery methods

**Parent group intervention methods: Sharing**,** normalization**,** and mutual support**. All participants were in favor of delivering the intervention in a group format in both Delphi rounds. Some expressed their views in the open-ended comments on the benefits of being in a group, such as fostering diversity, having in-depth discussions, and creating a safe space for sharing (*n* = 9), validating and normalizing each other’s experiences (*n* = 8), enabling the creation of connections and a sense of belonging (*n* = 4), allowing everyone to be considered and to have a place as parents (*n* = 3), and facilitating access to intervention compared to an individual intervention (*n* = 2).

**Number of parents.** All participants in the first Delphi spontaneously shared in the open-ended comments that intervention groups should be small. More specifically, they indicated the group should include between 4 (two participants suggested this number; 5 families/group: seven participants; 6 families/group: four participants; 8 families/group: four participants) to a maximum of 10 (four participants suggested this number) families. All participants in the second questionnaire (100%) reported agreement with having groups of 4 to 10 participants.

**Participation of both parents in the group.** In order to support both parents and promote co-parenting, participants were asked their opinion regarding the mandatory participation of both parents from the same family in the intervention group (yes or no question). Among participants, 19 (90.5%) were against having both parents. In the second questionnaire, 100% of participants agreed with offering the choice for the participation of a second parent or another significant adult for the family (e.g., grandparent) in the intervention.

**Transdiagnostic group.** When asked, “Are you in favor of a group intervention that would include a diversity of pediatric cancer diagnoses?”, 16 parents said they were in favor, while 5 parents said they were against it. Several participants spontaneously commented on the contributions and positive effects of transdiagnostic groups as presented in Table 5. In the second questionnaire, participants were able to indicate whether they were in favor of transdiagnostic groups, if this characteristic of the program was clearly mentioned to parents upon invitation to the program, and if groups were formed based on common service needs, particularly in relation to the stage of the disease, the extent of treatment, and the prognosis. All participants were then in favor.

**Facilitator Dyad.** In both questionnaires, all participants expressed support for program delivery by a clinician-parent dyad whose child had already been diagnosed with cancer. All participants spontaneously shared their contributions to this program characteristic (see Table 3 for themes). Participants also commented on the desired characteristics of the parent facilitator as well as those desired of the clinician facilitator (thematic analysis presented in Table 4).

**Table 4:** Characteristics perceived as important in program facilitators

	Theme	R1	Example quotes
Parent Facilitator Characteristics	Taking step back from own experience	18	“Not letting their own story take up too much space”; “The parent should have gone through various situations in order to better understand the participating parents (e.g., chemo, gaffe [transplant], radiation, surgery, post-treatment side effects, etc.), but without revealing too much about themselves unless it is beneficial to the group or to a particular parent.”
	Empathy/caring	15	“Compassionate empathy.”
	Capacity to listen	13	“Being attentive, just like the clinician…”
	Strong capacity for introspection	12	“Capacity for introspection…”
	Ability to set boundaries	7	“Not too focused on their own personal experiences, as they are there to support others.”
	Open-mindedness	6	“An open mind.”; “The same as the professional, with the addition of being open to sharing their own experience, while also making space for participants.”
	Resilient and emotionally stable	6	“Capacity to live difficult situations with other families with resilience.”; “”The parent should be able to maintain some emotional distance (as much as possible) in order to truly act as a helping parent, and not another parent to help.”
	Communication skills	5	“A good sense of communication.”; “An ease in expressing their personal experience in an educational way.”
	Not in an active grieving process	3	“I wonder whether a parent who has lost their child could manage to participate, and whether it would even be appropriate. I don’t think so—but of course, it always depends on the parent.”; “Not being in a mode of grievance…”
	Understands their role and is available to commit to it	3	“Someone who clearly understands their role and is there to give back, not to continue their own healing process.”
	Strong analytical skills	2	“Strong analytical mind.”
Clinician Facilitator Characteristics	Experience/skills/training in cancer and mental health	21	“The facilitator must be solid in the face of the difficult experiences of the parents and their children and must have experience in oncology. In my case, at the beginning of my journey as a parent of a child with cancer, I encountered psychological or psychosocial professionals who were overwhelmed by the reality I was living. I could see in their eyes that they found my situation terrible and that it frightened them. They had few concrete tools to help me cope with my reality. I eventually received the support I needed from a psychologist specialized in oncology. She understood what I was going through, was not afraid of it, and gave me concrete strategies to manage the situation. She helped me a lot.”; “Good knowledge of available resources.”
	Empathy, compassion, respect, caring	19	“This facilitator must also have listening and empathy skills.”; “respect and empathy.”
	Listening skills	9	“A good listener.”; “active listener.”
	Organized, skills in group facilitation, strong communication	8	“Ability to manage group dynamics during facilitation.”; “”Having good experience facilitating online support groups.”; “”Professionals who have already facilitated groups or who have training in group facilitation. Ideally, they should come from the psychosocial field (e.g., psychologist, social worker, psychoeducator).”
	Humility and awareness of one’s limits	7	“…I also believe that the professional should demonstrate genuine humility and not come from a ‘know-it-all’ position.”
	Open-mindedness and non-judgmental attitude	5	“Openness”; No judgement. Does not impose their ideas.”
	Ability to self-reflect	3	“Able to take a step back and see the bigger picture.”
	Energetic and cheerful	3	“Smiling, cheerful…”
	Ability to connect and build relationships easily	2	“”Able to easily build rapport and establish a trusting relationship.”
	Leadership and ability to set limits	2	“Someone who is comfortable setting boundaries with a group and has the leadership skills needed to guide the group to completion.”; “ “…They must be able to stay on course, as some parents tend to take up a lot of space in groups.”; “
	Ability to manage difficult situations	1	“Someone who can guide the discussion while truly making space for the participants, with the ability and sensitivity to identify the more vulnerable ones.”

**Online Delivery.** Based on the capacity of the community organization, which covers all regions of the province, it was determined in advance that the program would be delivered online. To ensure the program’s suitability as delivered online, and offer levers as needed for online adaptation, participants were asked two questions: “Do you see any drawbacks to the online format?” and “What could we do to facilitate online participation?” Respondents detailed several elements that could represent obstacles or facilitators (see Table 3) and essential information for implementing levers and ensuring the quality of the online program.

### Program/curriculum structure

**Preliminary meeting.** In the open-ended questions, 15 participants spontaneously gave feedback on including an individual preliminary meeting before the start of the group program. A question was asked about their opinions on what the objectives of this meeting should be. Five themes are presented in Table 3, allowing us to define the objectives and actions to be taken during this meeting. In the second questionnaire, these elements were proposed to participants for the preliminary meeting: Clearly explaining the program to parents (process, implications, etc.), meeting parents’ expectations and sharing those of the facilitators (program objectives; participant commitment/participation, etc.), ensuring a good match between what the program offers and the needs identified by parents, redirecting them if necessary, and assessing parents’ psychological health and identifying needs. All participants (100%) were in favor.

**Number**,** frequency**,** and duration of intervention sessions.** The number of sessions preferred by many participants was 5 to 7 sessions (*n* = 13). Five participants suggested less than 5 sessions, and 3 participants suggested 8–10 sessions. Nine individuals suggested that the sessions should be once a month, 7 suggested that it should be twice a month, 5 suggested once a week, and 3 suggested varied frequencies. As for the duration, 13 individuals reported preferring sessions lasting a maximum of one hour, 6 reported sessions of 1.5 h, 1 indicated less than one hour, and 1 suggested 2 h or more. There was no consensus regarding the time slot that the program should be offered, despite a preference for weekday evenings, and three participants mentioned that this should be adapted according to the realities of the parents for each group. During the second questionnaire, the following proposal was made to the participants: 8 sessions (including the preliminary meeting), 1 session every two weeks, 1 h per session, one weekday evening. 13 participants responded that they agreed completely, and 5 participants mentioned that it would be dense and suggested increasing the duration, in particular opting for sessions of an hour and a half (“1 hour per session seems tight. If we want exchanges… 10 minutes/person is already almost 2 hours. We don’t want people to feel rushed. I would suggest 1.5 hours because it could easily overflow.”).

**Program-related materials for participants**. Three types of materials to be offered to parents during the program were proposed to participants for the first Delphi: (1) a handbook of services and resources available in the context of pediatric cancer; (2) a participant workbook outlining the program’s timeline, session by session, and a reminder of the strategies discussed in the group; and (3) exercises to be completed outside of the session (available in the participant workbook). All participants were in favor of these three options. Participants agreed that the handbook of services and the participant workbook should be available before the program began (*n* = 13 participants). Seventeen participants mentioned that the exercises outside of the sessions should be optional.

During the second Delphi, participants were informed that the program would provide a comprehensive overview of the services and resources available from the start of the program and a participant workbook summarizing the content of each session, as well as extra activities to be completed outside of sessions. All 18 participants fully agreed.

**Measures and actions to implement in case of distress.** Participants were asked their opinion about the measures to implement for parents in case of greater support needs, who might require more intensive and individualized assistance than what is offered by the group. All participants (100%) agreed with this measure and specified various themes allowing for the identification of actions that it should include (see Table 3).

During the second questionnaire, participants were informed of the actions that would be prioritized in this measure: informing the community pediatric cancer organisation who supports the parent, offering individual time to the parent with the clinician-facilitator, proposing resources and solutions (previously identified), and liaising with other service partners in this regard. Seventeen participants fully agreed, and one person suggested adding a contact person (e.g., family member, friend, spouse) that was agreed upon between the program clinician and the participating parent who could be contacted in case of emergency.

**Therapeutic approaches.** A literature review on the most effective mental health and parenting interventions in the context of cancer was conducted by a specialist librarian, which allowed for general suggestions for participants, namely, to know which interventions they considered to be the most effective. Table 3 reflects participants’ preferences, with the acceptance and commitment therapy (ACT) approach being the most common.

Participants also clarified certain aspects during open-ended questions (one participant per theme): The use of psychoeducation to teach strategies, the use of an evidence-based approach, sharing and normalizing emotional experiences, the use of activities to encourage the application of proposed strategies, fostering a balance between sharing experiences and psychoeducation, and providing concrete examples.

In the second Delphi questionnaire, participants were asked about the suitability of the theoretical approach, ACT, which was the preferred choice in the first questionnaire, while ensuring adaptation to the context of the trauma and a focus on learning concrete coping strategies. All participants were in favor of this approach without reservation. Following suggestions in the Delphi 1 questionnaire, participants were asked if adding an “emotional journaling” component at the beginning and end of the program session would be a preferable strategy to create a space for parents to “check-in” and identify their experienced emotions. All participants agreed, but two mentioned that completion of the journal should be personal and optional.

## Results: focus group

**Fig. 2:**
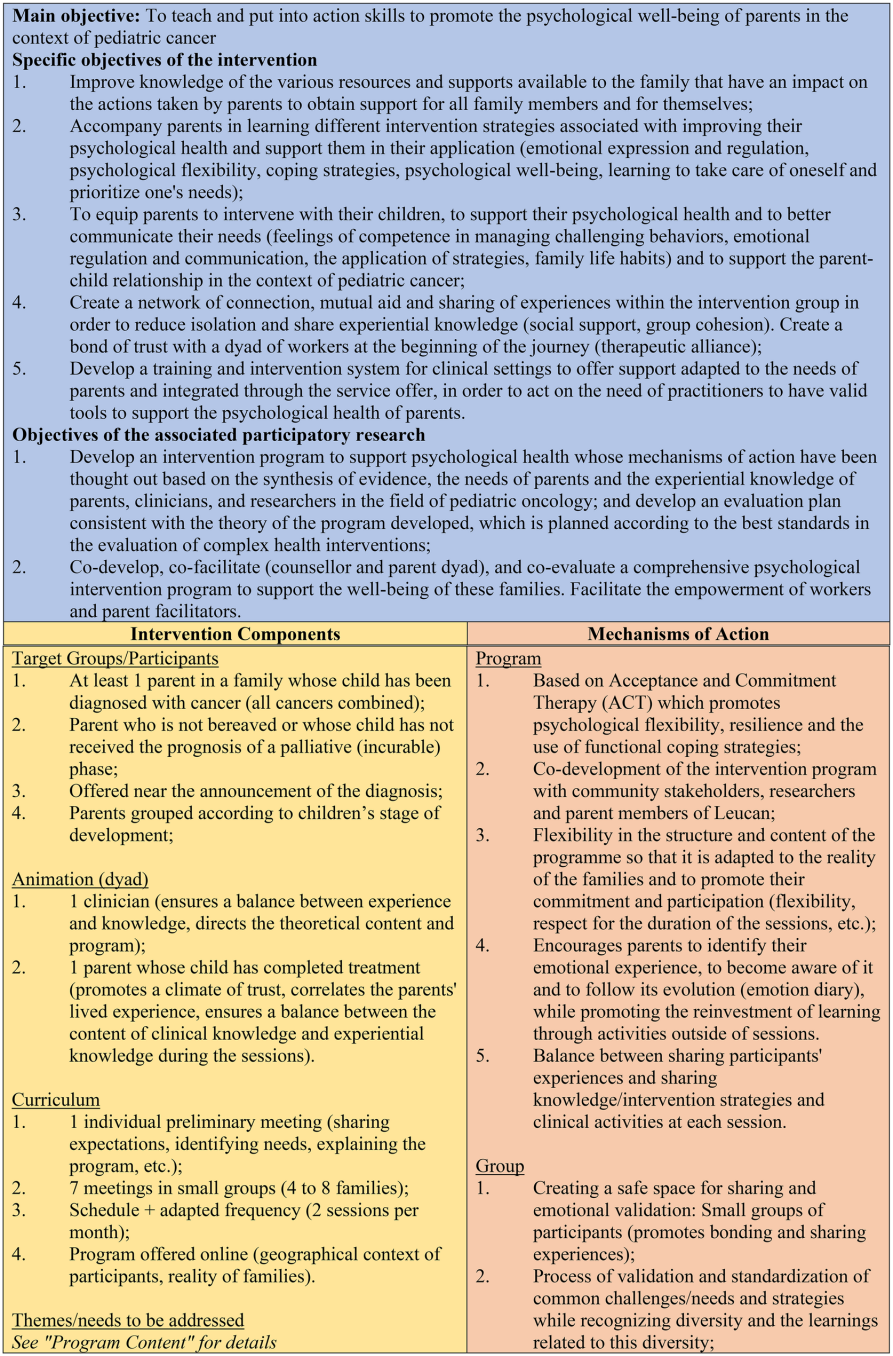

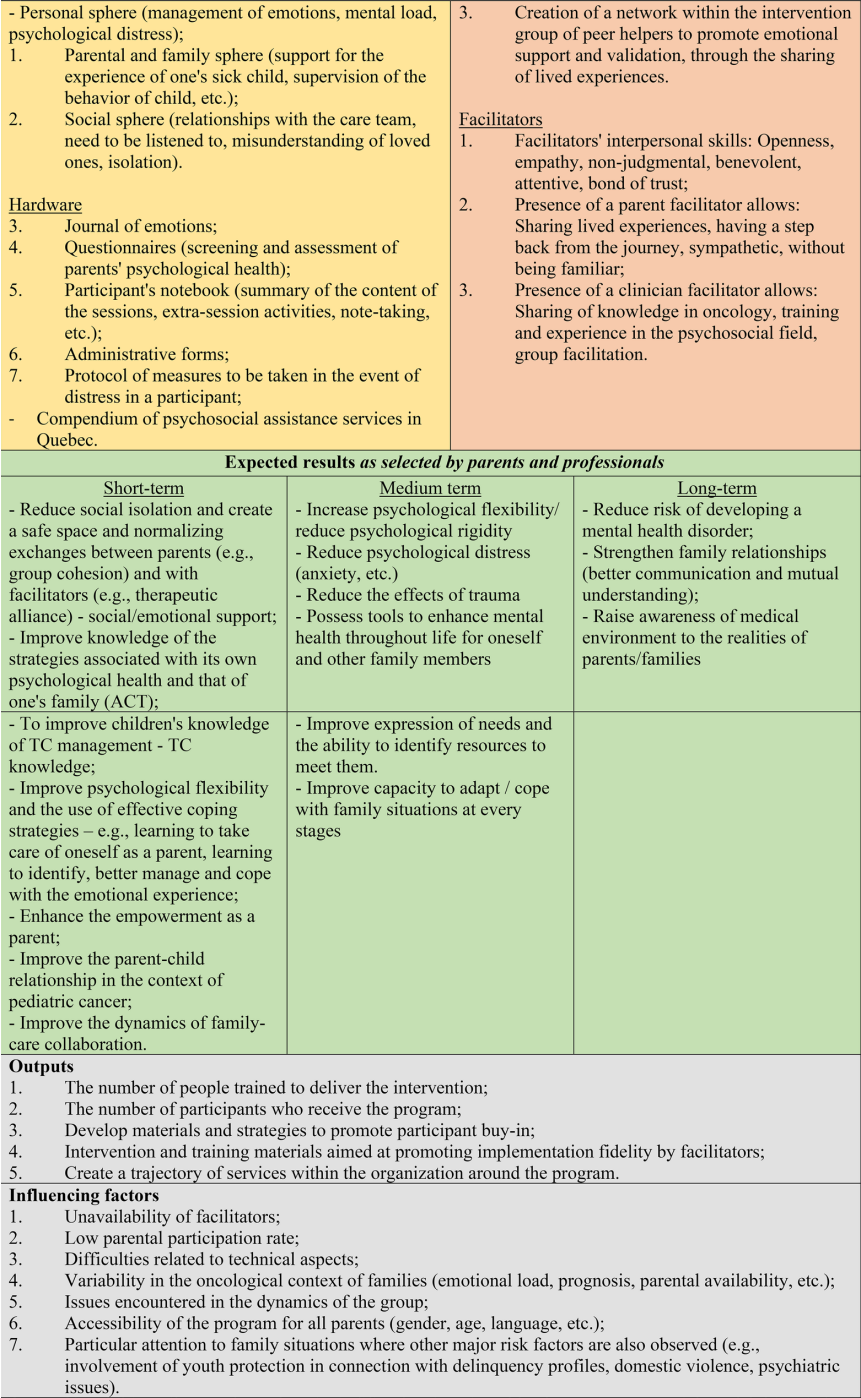
Logic model of the program “Me for Us”

The results of the focus group allowed for validation of the model and are presented and summarized in Table 5. Table 6 presents each session of the final program and Fig. 2 presents the final logic model of the intervention, co-constructed and validated through the modified Delphi and focus groups.

**Table 5:** Summary of results from focus groups

Theme	Sub-theme	Un	N1 = 5	N2 = 4	N3 = 5	Value Total		
						Fr	%	Ngr
Objectives	The objectives are clear	3	1	3	2	6	42,9%	3
	Reformulate objectives in a way that is more accessible to parents	3		1	2	3	21,4%	2
	Add the objective “Train professionals”	2	1	3		4	28,6%	2
	Creating a “social network” or peer helpers should not be an objective	6	1	4	4	9	64,3%	3
	The objective “Filling a gap in services continuum” should be added	5	3	1	1	5	35,7%	3
Session Themes	Emphasize the parent-child relationship	5	1		5	6	42,9%	2
	Validate proposed topics	4	4	3		7	50,0%	2
	Specify what relates to the diagnosed child versus siblings	1		1		1	7,1%	1
	Themes are interconnected, should be addressed in all sessions	3	1		5	6	42,9%	2
	Do not mix themes in sessions	1			1	1	7,1%	1
	Validate not addressing financial aspects as a direct theme	2	2			2	14,3%	1
	Address management of the extended family network	1		2				
Participants	Validate the exclusion criteria: parents in palliative care	6	4	1	2	7	50,0%	3
	Validate the exclusion criteria: bereaved parents	4	1	2	2	5	35,7%	3
	Validate inclusion of caregivers other than biological parents	1		3		3	21,4%	1
	Allow flexibility regarding time since diagnosis (3 months)	7	2	2	4	8	57,1%	3
Group Formation Modality	Validation: Treatment intensity	5	3	1	2	6	42,9%	3
	Validation: Child’s. age (developmental stage)	4		3	5	8	57,1%	2
	Validation: Prognosis	3		1	2	3	21,4%	2
	Validation: Group of mixed diagnoses	3		2	2	4	28,6%	2
	Groups of all ages (remain flexible)	2		1	1	2	14,3%	2
	Groups of all prognoses (remain flexible)	1		1		1	7,1%	1
Session Modality	Suggestion to add a 9th individual session	1		3		3	21,4%	1
	Suggestion to have a group follow-up session (booster)	5		4	2	6	42,9%	2
	Suggestion to have longer sessions (1.5 h)	1		1		1	7,1%	1
	Suggestion to have groups of 4–6 families (4–6 parents if 1 per family; 8–12 if 2 per family)	2			3	3	21,4%	1
Materials	Protocol in case of distress to be used throughout the program	8	5		5	10	71,4%	2
	Offer support for completing questionnaires before and after the program	2		4	2	6	42,9%	2
	Completion of questionnaires to systematically screen emotional health during the program	1		2		2	14,3%	1
	Remind families of available services + ACT tool summary at the end	4	2	2	2	6	42,9%	3
	Suggestion to have testimonial videos at the beginning (hope and resilience)	1		4		4	28,6%	1
	Validate participant workbook	1	1			1	7,1%	1
	Suggestion to make the emotional journal optional	1			1	1	7,1%	1
Preliminary Session	Validate content and objectives of the meeting	3	2		1	3	21,4%	2
	Suggestion to have the presence of both facilitators	1			2	2	14,3%	1
Facilitation	Structure and manage time to ensure equitable sharing	6	2	4	1	7	50,0%	3
	Support the parent-facilitator	2		2	1	3	21,4%	2
	Validate co-facilitation as a dyad	3		1	3	4	28,6%	2
	Ensure no helper-recipient relationship between facilitators	1	1			1	7,1%	1
	Validate facilitator characteristics	3	2		1	3	21,4%	2
Precisions on Mechanisms of Action	Explain the program’s co-production history/process to parents (support motivation and sense of fit from the start)	1		3		3	21,4%	1
	Connect parents to the continuum of services	2	2			2	14,3%	1
	Provide concrete tools and tips (based on ACT)	2	2			2	14,3%	1
Short-term Effects	Reduce isolation	3	2	4	1	7	50,0%	3
	Normalize experiences	7	3	2	3	8	57,1%	3
	Learn psychological flexibility and coping tools	4	1	2	2	5	35,7%	3
	Learn to take time for oneself and prioritize	3		2	1	3	21,4%	2
	Identify, release, and better manage emotions	3		1	1	3	24,1%	3
	Support a better family dynamic and life	4	1		2	3	21,4%	2
	Increase power to act (empowerment)	2	1		1	2	14,3%	2
	Improve collaboration between parent and professional	1	1			1	7,1%	1
Medium to Long-term Effects	Manage trauma effects	2	1		1	2	14,3%	2
	Integrate psychological distress management tools	4	1	1	2	4	28,6%	3
	Adapt the family system	4		1	3	4	28,6%	2
	Sensitize health professionals to family realities	1			1	1	7,1%	1
	Create a mutual support network (as needed and voluntary)	6	1	2	5	8	57,1%	3
	Improve mental health of family members	1			1	1	7,1%	1

1: focus group 5 clinicians; 2: first focus 4 group parents; 3: second focus 5 group parents

Un : The number of meaning units categorized in each sub-theme; Fr : Number of times named in a group; % : percentage of participants who named this theme in a group; Ngr: Number of groups that named this sub-theme

**Table 6:** Program session content and objectives

Content	Session	Specific Objectives
Framing the Intervention, Individualized Support & Reducing Barriers	Preliminary individual session & Individual weekly call	- Build engagement/connection; - Conduct continuous assessments on psychological well-being/family situation; - Insure program’s adequacy and practical support needs; - Implement additional measures, references and the distress protocol* (apply it if necessary).
Bases of ACT: Taking Care of my Own Emotional Well-being	Session 1	- Knowing each other and settings the base - Information support: the Guide of services* - Identify and express feelings and emotions (emotional journaling*) - Recognize automatic responses to difficult thoughts and emotions - Understand how being fused with thoughts/emotions can influence reactions
	Session 2	- Identify personal values (individual and as a parent) - Recognize behaviors that distance us from what really matters - Understand anxiety responses and reactions through the lens of our lived experiences
	Session 3	- Understand and apply the six core processes of ACT/psychological flexibility: learning to accept thoughts/emotions and commit to value-driven actions, both in the context of pediatric cancer and as an individual.
Using ACT to Navigate the Emotional Dance Between Parents and Children	Session 4	- The cycles/steps of the “emotional dance” parent-child - Recognize situations where we are caught in a dynamic of automatic emotional cycle with our child - Identify our automatic reactions facing our child difficult emotions and behaviors – functional assessment and take a step back with ACT
	Session 5	- Identify effective strategies to teach our child self-regulation and emotional well-being skills - Develop strategies to manage emotional danse and conflicts with our child in difficult moments
Managing Challenging Behaviors	Session 6	- Understand how challenging behaviors emerge, develop and their functions - Identify and apply functional interventions
Conclusion	Session 7	- Integrative Exercise: Applying ACT Principles in Daily Parenting Challenges - Redefining our group: what next for parents as a group

The final stage of the co-creation process for the intervention logic model consisted of three focus groups aimed at validating the different components of the logic model, selecting the effects of the program with participants based on the program theory presented, and gathering concluding remarks for finalizing the model. Analysis of the integrated data from the three focus groups identified 10 general themes (composed of subthemes) to finalize the model and are presented in Table 5. Globally, for objectives, stakeholders reported that the objectives were valid and clear although there was a need to make the terminology more accessible to parents. Participants in all three focus groups mentioned that the goal of creating a social network (or peer supporters) should be removed because they felt that the program should retain the goal of group therapy to support the well-being and psychological flexibility of parents and not to become a support group where parents might feel compelled to support others and maintain long-term bonds. While they felt that the group process within the program was highly relevant, they noted that it should not become a support measure where parents taking part in the intervention are expected to become peer supporters and that there are expectations of them in that regard. Participants also called for two additional objectives: To train clinicians and fill a gap in the services continuum. The topics to be covered to meet these objectives were validated in the three focus groups and participants testified to the importance of dedicating time in the intervention to the emotional health of parents and their sick child and the impacts on their relationship. They agreed that the program can support parents to work on this relationship in the context of treatments and illness.

The focus group participants also validated the inclusion and exclusion criteria and the principles for forming the groups, including the importance of basing the groups on the extent of treatment, the child’s developmental phase and the prognosis. Program delivery modalities were also validated, although it was advisable to extend the sessions of the program to a duration of 1.5 h. Two suggestions were made for the addition of sessions: an individual meeting to conclude the program and a follow-up group meeting after the end of the program. The number of participants per group had been set to 4 to 6 families if only one parent participated and 8 to 12 parents if two parents participated. The program material was also validated, which reflects the importance of the use of the distress support protocol, questionnaires, portrait of services and participants’ workbook throughout the program. It was also suggested to add capsules of testimonials of parental participation in the program. Program facilitation by a dyad of facilitators was also validated, as well as the importance of equitable delivery by the two facilitators and essential characteristics for both. The program’s mechanisms of action were also accepted by the focus groups and participants stressed the importance of clearly mentioning to parents the history of co-construction of the “Me for Us” program so that parents can understand upon invitation that the program is relevant and sensitive to the real needs of families. They also reiterated the importance of connecting parents participating in the program to other services in the continuum of services present in the health, social, and community services networks and to ensure that the tools offered in ACT are concrete and practical in everyday life.

Finally, the focus groups made it possible to target with stakeholders the effects of the “Me for Us” program after they had been able to grasp the objectives and mechanisms of action of the program, co-constructed with them. Thus, at the end of the focus group, participants were able to reflect together on the short- and medium-term effects that the program should aim for. Details of the targeted effects are presented at the end of Table 5.

## Discussion

Documenting the early stages of intervention development prior to conducting efficacy trials provides a clear rationale for the evaluation process and enhances the likelihood of successful, sustainable implementation [11]. This study emphasizes the critical impacts of collaborative partnerships between families, services stakeholders, and researchers in making shared decisions throughout the intervention development phase, incorporating the expertise of both those who deliver and those who receive pediatric cancer services. By centering the voices of those most directly impacted, this research aims to establish a foundation for interventions that are not only more person-centered and responsive, but also better equipped to address persistent challenges related to access, cost, and the lack of standardized psychosocial care for families of children diagnosed with cancer.

### The “Me for Us” group parental intervention: a community- and ACT-based program

The present iterative mixed-method study allowed us to co-construct with parents, clinicians, researchers and community-based services managers a logic model for an intervention specifically designed to address the emotional health needs of parents, as well as promote parents’ ability to support the emotional well-being of their child and other family members.

The final logic model of the “Me for Us” intervention outlines a structured, manualized, eight-session (one individual preliminary session and seven group sessions) program designed to address the overarching objective of teaching and activating skills that promote the psychological well-being of parents facing a pediatric cancer diagnosis. More specifically, the program aimed to help parents: (1) take time for themselves and learn to prioritize their own needs; (2) identify and manage their emotions more effectively; (3) reduce isolation and normalize their experiences through in group peer support; (4) integrate tools that foster psychological flexibility and effective coping strategies; (5) support their child’s emotional well-being and strengthen their relationship with the child undergoing treatment; and (6) increase their knowledge of available services and resources, their use, enhance collaboration with healthcare providers. In addition, the stakeholders participating in this intervention co-development project expressed the hope that the program would ultimately develop a training and intervention system for clinical settings to offer support adapted to the needs of parents and be integrated through the services offered, in order to act on the need of workers to have access to valid tools to support the psychological health of parents. Thus, through this participatory project, the team of collaborators hopes that the intervention can generate a social transformation rooted in everyday life services, ranging from the training of workers for an approach that includes support for parents very early on their children services trajectory the concrete deployment of a permanent parent intervention in services.

The “Me for Us” program is delivered by a dyad of facilitators, an approach designed to partially address embedded structural access barriers to mental health professionals. First, the professional facilitator may hold various roles, positions, or professional titles (e.g., nurse, psychologist, social worker, therapist) within the community organization and is not required to have a specific professional designation. This flexibility was identified by the advisory committee as a critical feature, especially given the limited availability of trained psychologists within the Quebec health and social services system. As a next step within the CBPR process, and more specifically within the development and evaluation of the “Me for Us” program, the co-development of structured training for facilitator dyads is planned. This training will constitute a core mechanism for ensuring high-quality intervention delivery, alongside the careful selection of candidates suited to this type of intervention (see Table 5 for a description of the desired competencies and qualities). The facilitator training will be developed by the advisory committee based on the findings of the present study and will follow a co-development process similar to that used for the intervention itself. A foundational principle of this training will be to ensure that it can be delivered in public and community organisation settings using accessible pedagogical and supervision modalities that minimize burden in terms of time, cost, and travel, while maintaining rigor and high training quality.

### Theoretical foundations

From a theoretical point of view, the development of “Me for Us” is based on both the foundations of CBPR and the clinical processes of ACT. This integrated approach makes it possible to respond in a contextualized way to the emotional and social needs of parents of children with pediatric cancer, a reality marked by uncertainty, psychological distress and the impossibility of changing certain living conditions [9]. The intervention thus offers a flexible and value-based framework, adapted to the diversity of diagnoses and the heterogeneity of parental experiences.

Second, parents, clinicians, managers, and researchers involved in this co-construction process unanimously expressed the importance of building a group program structured around ACT processes. ACT seeks to transform an individual’s relationship with difficult thoughts and emotions, which is particularly important in contexts where they reflect an objective and unchangeable reality (e.g., “My child has a life-threatening condition”, “My family would probably suffer”). Having this shift in perspective is particularly relevant in pediatric oncology, where situations are not always changeable, but suffering can be alleviated through better emotional regulation and increased engagement with individuals’ values and what really matters to them in this context (e.g., Being the parent that I want to be for my child in this context of adversities). Acceptance has been shown to be a key coping strategy associated with reduced distress in parents of children diagnosed with cancer [26]. ACT skills allow parents to better cope with intrusive thoughts, guilt, or fear, which are frequently observed in the caregiving role in the context of pediatric cancer, while strengthening their ability to intentionally choose how they want to be as parents, despite adversity. Byrne et al., [27] have shown that ACT is a promising intervention to support parents of children living with physical or psychological difficulties. Other studies confirm that parental psychological flexibility is linked to better adaptation and lower levels of internalizing and externalizing behaviors in children [28]. Improved caregiver coping is, in turn, associated with more effective communication with the child diagnosed with cancer [29]. In addition, engagement in valued activities and acceptance of painful experiences appear to be key mechanisms in reducing psychological distress among family caregivers [30, 31].

### Group process

Another essential mechanism of change within the intervention is the group process itself. It was very important for our stakeholders that the group was formed with a small number of parents, between 4 and 8 families maximum, as a small group setting fosters a supportive environment where shared experiences reduce isolation and normalize emotional responses in a secure and connected space. Within this context, accepting discomfort becomes not only a personal endeavor but also a collective experience, reinforced through interactive exercises and metaphors grounded in everyday narratives. During the co-production of the logic model of “Me for Us,” it was very clear for participants that there was a need for a small group, deep connection across the participants and a strong therapeutic alliance with the group facilitators.

Several studies emphasize that the effectiveness of group interventions is largely based on the dynamics that emerge within the group itself [32]. These dynamics, called group processes, refer to the relational and psychological mechanisms that promote change and influence therapeutic outcomes. Some of these processes play a key role, such as the opportunity to express and receive interpersonal feedback, the sharing of personal experiences, targeted interventions by the facilitator, and the sense of unity and solidarity among members – known as group cohesion. The latter is widely recognized as one of the most powerful levers in group therapy [33].

### Dyad of clinician and parent facilitators

Another targeted mechanism of change in the “Me for Us” program lies in the fact that the group intervention is co-facilitated by a trained mental health professional and a parent partner with a lived experience of the pediatric oncology journey, which greatly strengthens other therapeutic mechanisms of action. This co-facilitation enriches group processes, such as cohesion, emotional resonance, mutual validation, and self-disclosure. The parent facilitator also represents a peer by integrating both the program’s expertise and embodying experiential knowledge. Other parents can feel understood and validated by someone with a shared experience. The presence of the parent partner facilitates identification with the group, creates a form of realistic hope, and fosters a climate of trust encompassing authentic sharing. At the same time, the professional ensures a safe environment, supports complex emotional processes, and mobilizes targeted interventions based on the group’s needs.

### Connection with information and services

A critical aspect of the “Me for Us” program is that in addition to offering support in a group format based on parents’ individual needs, it has valuable connections with organizations that are implicated directly in the community (i.e., the large provincial pediatric cancer organization). This both enriches the intervention program itself and offers consistent and integrated support and information for parents through ongoing screening of needs. Having a community organization involved in the research process can serve as an effective way to provide feelings of familiarity for parents, as well as offer them a sense of security. Furthermore, in a program such as the present one, organization clinicians can act as an important liaison between parents and the research team.

### Limitations

We recognize that, while we strived for engagement of multiple knowledge users throughout the project, the sample size may not allow for representative views of all parents and professionals in pediatric cancer. We adhered to Delphi method sample size recommendations and presented participants with information based on prior pediatric cancer studies cumulatively spanning hundreds of participants. We note that none of the caregiver participants of the current study identified as male. The mental health vulnerability of mothers of children diagnosed with cancer has been extensively studied and has previously been the focus of dedicated interventions. There is a dearth of literature on the mental health of fathers of children diagnosed with cancer and their involvement in biopsychosocial outcomes of children diagnosed with cancer and their specific psychosocial support needs [34–36]. Findings from the general population highlight that men and women respond to severe stressors differently, with unique mental health symptom presentations and support preferences. Future research specifically focusing on the psychosocial support needs of fathers is warranted. We also note that “Me for Us” is not intended to replace existing psychosocial supports, but rather to complement the array of psychosocial resources currently being offered to parents. Furthermore, although “Me for Us” was designed based on majority feedback from participants and many components were endorsed by all (100%) of participants, parents’ needs may vary. Some parents may prefer individual therapy approaches, and these should also be offered.

## Conclusion

There is a definite need to support the psychological wellbeing of caregivers of children diagnosed with cancer. Intervention development should be rigorous and aligned with parents and clinicians’ needs and preferences. To this end, “Me for Us,” grounded in the principles of CBPR and ACT, creates a novel, participatory, and context-sensitive approach for caregivers of children diagnosed with cancer. Consistent with the co-development and co-evaluation model plan, the next step of pilot testing this intervention with parents of children diagnosed with cancer is underway. Ultimately, this intervention may serve as a model for much needed psychosocial support programs for caregivers of children faced with other pediatric chronic illnesses, and the logic model of the present program may be able to inform other types of program development (e.g., for siblings or for children who have the diagnosis themselves).

## Data Availability

The datasets used and analysed during the current study are available from the corresponding author on reasonable request.

## References

[1] Koumarianou A, Symeonidi AE, Kattamis A, Linardatou K, Chrousos GP, Darviri C. A review of psychosocial interventions targeting families of children with cancer. Palliat Support Care. 2021;19(1):103–18. 10.1017/S1478951520000449. 32613930 10.1017/S1478951520000449

[2] Ogez D, Péloquin K, Bertout L, et al. Psychosocial intervention programs for parents of children with cancer: a systematic review and critical comparison of programs’ models and development. J Clin Psychol Med Settings. 2019;26(4):550–74. 10.1007/s10880-019-09612-8. 30806900 10.1007/s10880-019-09612-8

[3] Sultan S, Leclair T, Rondeau E, Burns W, Abate C. A systematic review on factors and consequences of parental distress as related to childhood cancer. Eur J Cancer Care. 2016;25(4):616–37. 10.1111/ecc.12361 PMC5049674 26354003

[4] Hu X, Grosse SD, Han X, Marchak JG, Ji X. Mental health care utilization among parents of children with cancer. JAMA Netw Open. 2024;7(4):e244531–244531. 10.1001/jamanetworkopen.2024.4531. 38564218 10.1001/jamanetworkopen.2024.4531 PMC10988353

[5] Rodriguez EM, Dunn MJ, Zuckerman T, Vannatta K, Gerhardt CA, Compas BE. Cancer-related sources of stress for children with cancer and their parents. J Pediatr Psychol. 2012;37(2):185–97. 21841187 10.1093/jpepsy/jsr054 PMC3282279

[6] Van Warmerdam J, Zabih V, Kurdyak P, Sutradhar R, Nathan PC, Gupta S. Prevalence of anxiety, depression, and posttraumatic stress disorder in parents of children with cancer: A meta-analysis. Pediatr Blood Cancer. 2019;66(6):e27677. 10.1002/pbc.27677. 30816008 10.1002/pbc.27677

[7] Desjardins L, Solomon A, Shama W, Mills D, Chung J, Hancock K, Barrera M. The impact of caregiver anxiety/depression symptoms and family functioning on child quality of life during pediatric cancer treatment: from diagnosis to 6 months. J Psychosoc Oncol. 2022;40(6):790–807. 35016592 10.1080/07347332.2021.2015646

[8] Kearney JA, Salley CG, Muriel AC. Standards of psychosocial care for parents of children with cancer. Pediatr Blood Cancer. 2015;62(S5):S632–83. 26700921 10.1002/pbc.25761 PMC5066591

[9] Lewandowska A. The needs of parents of children suffering from cancer-continuation of research. Child (Basel). 2022;9(2):144. 10.3390/children9020144. 10.3390/children9020144 PMC8870376 35204865

[10] Scialla MA, Canter KS, Chen FF, Kolb EA, Sandler E, Wiener L, Kazak AE. Delivery of care consistent with the psychosocial standards in pediatric cancer: Current practices in the United States. Pediatr Blood Cancer. 2018;65(3):e26869. 10.1002/pbc.26869 PMC5766412 29080381

[11] Skivington K, Matthews L, Simpson SA, Craig P, Baird J, Blazeby JM, Boyd KA, Craig N, French DP, McIntosh E, Petticrew M, Rycroft-Malone J, White M, Moore L. A new framework for developing and evaluating complex interventions: update of Medical Research Council guidance. BMJ (Clinical Res ed). 2021;374(n2061). 10.1136/bmj.n2061. 10.1136/bmj.n2061 PMC8482308 34593508

[12] Rosa WE, Santos J, Agbeko AE, Barksdale CL, Carvajal S, Dillard D, Pérez-Stable EJ. Community-based participatory research: a lifeline to achieve people-centered care. Front Public Health. 2025;13:1693459. 10.3389/fpubh.2025.1693459. 41473713 10.3389/fpubh.2025.1693459 PMC12746848

[13] Wallerstein N, Duran B, Oetzel JG, Minkler M, editors. Community-based participatory research for health: Advancing social and health equity. Wiley; 2018.

[14] Rivard M, Tremblay J, Mestari Z, Desjardins L, Bergeron É, Lefebvre C. (2025). Survey of families’ psychosocial needs in the context of pediatric cancer: a first step toward the participatory development of a group intervention. Journal of Psychosocial Oncology. 43(4):496–512. 10.1080/07347332.2024.2404570 10.1080/07347332.2024.2404570 39283063

[15] Chen, H.-T. (2015). Practical program evaluation: Theory‑driven evaluation and the logic model (2nd ed.). SAGE Publications.

[16] McLaughlin JA, Jordan G, B. Using Logic Models. In: Kathryn E, Newcomer KE, Hatry HP, Wholey JS, editors. Handbook of Practical Program Evaluation. Wiley; 2015. pp. 62–87. 10.1002/9781119171386.

[17] Custer, R. L., Scarcella, J. A., & Stewart, B. R. (1999). The modified Delphi technique — a rotational modification. Journal of Vocational and Technical Education, 15(2), 50–58 10.21061/jcte.v15i2.702

[18] Rivard M, Jacques C, Hérault É, Mello C, Abouzeid N, Saulnier G et al. (2024). An innovative and collaborative method to develop a model care and service trajectory for the assessment, diagnosis, and support of children with developmental disabilities. Evaluation and Program Planning. 104:102431. 10.1016/j.evalprogplan.2024.102431 10.1016/j.evalprogplan.2024.102431 38608392

[19] Beiderbeck D, Frevel N, von der Gracht HA, Schmidt SL, Schweitzer VM. Preparing, conducting, and analyzing Delphi surveys: Cross-disciplinary practices, new directions, and advancements. MethodsX. 2021;8:101401. 10.1016/j.mex.2021.101401. 34430297 10.1016/j.mex.2021.101401 PMC8374446

[20] Linstone HA, Turoff M. The delphi method. MA: Addison-Wesley Reading; 1975.

[21] Schurmans M-N, Charmillot M, Dayer C. (2014). Introduction du Dossier La restitution des savoirs [Introduction to the Dossier Restitution of knowledge]. Association internationales des sociologues de langue française (AISLF). https://journals.openedition.org/sociologies/4713.

[22] Robinson OC. Conducting thematic analysis on brief texts: The structured tabular approach. Qualitative Psychol. 2022;9(2):194. 10.1037/qup0000189.

[23] Rabiee F. Focus-group interview and data analysis. Proc Nutr Soc. 2004;63(4):655–60. 15831139 10.1079/pns2004399

[24] Ritchie J, Spencer L. Qualitative data analysis for applied policy research. Analysing Qualitative Data. London: Routledge; 1994. pp. 173–94. [A Bryman and RG Burgess, editors].

[25] Krueger RA, Casey MA. Focus Groups: A Practical Guide for Applied Research. 3rd ed. Thousand Oaks, CA: Sage; 2000.

[26] Compas BE, Bemis H, Gerhardt CA, Dunn MJ, Rodriguez EM, Desjardins L, Vannatta K. Mothers and fathers coping with their children’s cancer: Individual and interpersonal processes. Health Psychol. 2015;34(8):783. 10.1037/hea0000202. 25622077 10.1037/hea0000202 PMC4671390

[27] Byrne G, Ghráda ÁN, O’Mahony T, Brennan E. A systematic review of the use of acceptance and commitment therapy in supporting parents. Psychol Psychotherapy: Theory Res Pract. 2021;94:378–407. 10.1111/papt.12282 32406169

[28] Brassell AA, Rosenberg E, Parent J, Rough JN, Fondacaro K, Seehuus M. Parent’s psychological flexibility: Associations with parenting and child psychosocial well-being. J Context Behav Sci. 2016;5(2):111–20.

[29] Rodriguez EM, Murphy L, Vannatta K, Gerhardt CA, Young-Saleme T, Saylor M, Compas BE. Maternal coping and depressive symptoms as predictors of mother–child communication about a child’s cancer. J Pediatr Psychol. 2016;41(3):329–39. 26609183 10.1093/jpepsy/jsv106 PMC5013837

[30] Jansen JE, Haahr UH, Lyse HG, Pedersen MB, Trauelsen AM, Simonsen E. Psychological flexibility as a buffer against caregiver distress in families with psychosis. Front Psychol. 2017;8:1625. 29046649 10.3389/fpsyg.2017.01625 PMC5632725

[31] McCracken LM, Gutiérrez-Martínez O. Processes of change in psychological flexibility in an interdisciplinary group-based treatment for chronic pain based on Acceptance and Commitment Therapy. Behav Res Ther. 2011;49(4):267–74. 21377652 10.1016/j.brat.2011.02.004

[32] Burlingame GM, Strauss B, Joyce A. Change mechanisms and effectiveness of small group treatments. Bergin Garfield’s Handb Psychother Behav change. 2013;6:640–89.

[33] Burlingame GM, McClendon DT, Yang C. Cohesion in group therapy: A meta-analysis. Psychotherapy. 2018;55(4):384. 30335452 10.1037/pst0000173

[34] Bonner MJ, Hardy KK, Willard VW, Hutchinson KC. Brief report: Psychosocial functioning of fathers as primary caregivers of pediatric oncology patients. J Pediatr Psychol. 2007;32(7):851–6. 17426044 10.1093/jpepsy/jsm011

[35] Brody AC, Simmons LA. Family resiliency during childhood cancer: The father’s perspective. J Pediatr Oncol Nurs. 2007;24(3):152–65. 17475981 10.1177/1043454206298844

[36] Olsavsky AL, Benhayoun A, Lyman E, Franklin B, Ranalli M, Skeens MA. (2025). Paternal contributions to biopsychosocial outcomes of children with cancer: A scoping review. Pediatric Blood & Cancer, 72(1):e31752 10.1002/pbc.31752 10.1002/pbc.31752 PMC12101929 40339061

